# Extended dynamic range imaging for noise mitigation in fluorescence anisotropy imaging

**DOI:** 10.1117/1.JBO.25.8.086003

**Published:** 2020-08-20

**Authors:** Paolo Fumene Feruglio, Claudio Vinegoni, Ralph Weissleder

**Affiliations:** a Massachusetts General Hospital, Harvard Medical School, Center for Systems Biology, Boston, Massachusetts, United States; b University of Verona, Department of Neuroscience, Biomedicine, and Movement Sciences, Verona, Italy; c ITS Meccatronico Veneto, Vicenza, Italy; d Harvard Medical School, Department of Systems Biology, Boston, Massachusetts, United States

**Keywords:** optical microscopy, optical imaging, confocal imaging, fluorescence anisotropy, ratiometric imaging

## Abstract

**Significance:** Fluorescence polarization (FP) and fluorescence anisotropy (FA) microscopy are powerful imaging techniques that allow to translate the common FP assay capabilities into the *in vitro* and *in vivo* cellular domain. As a result, they have found potential for mapping drug–protein or protein–protein interactions. Unfortunately, these imaging modalities are ratiometric in nature and as such they suffer from excessive noise even under regular imaging conditions, preventing accurate image-feature analysis of fluorescent molecules behaviors.

**Aim:** We present a high dynamic range (HDR)-based FA imaging modality for improving image quality in FA microscopy.

**Approach:** The method exploits *ad hoc* acquisition schemes to extend the dynamic range of individual FP channels, allowing to obtain FA images with increased signal-to-noise ratio.

**Results:** A direct comparison between FA images obtained with our method and the standard, clearly indicates how an HDR-based FA imaging approach allows to obtain high-quality images, with the ability to correctly resolve image features at different values of FA and over a substantially higher range of fluorescence intensities.

**Conclusion:** The method presented is shown to outperform standard FA imaging microscopy narrowing the spread of the propagated error and yielding higher quality images. The method can be effectively and routinely used on any commercial imaging system and could be also translated to other microscopy ratiometric imaging modalities.

## 1 Introduction

Direct *in vivo* fluorescence intensity measurements are crucial for measuring fluorescence marker concentration or other intrinsic parameters, but they can be quite challenging due to the absence of an internal reference and the presence of various nonrelevant artifacts, e.g., tissue scattering and absorption, sample–detector path geometry, or fluctuations in the excitation source.^1^^,^^2^

Ratiometric fluorescence indicators are better posed than the fluorescence intensity ones and have been used extensively to measure for example changes of calcium ions concentrations,^3^ membrane potentials,^4^^,^^5^ and other parameters.^6^ Depending on the specific imaging modality, the fluorescence signal is typically measured via excitation or emission under different or the same conditions of polarizations and wavelengths, and the ratiometric quantity is directly or indirectly calculated by taking the ratio of the two measurements. The measurements are preferentially made simultaneously such that artifacts, if present, are counterbalanced. In a similar fashion, ratiometric redox fluorometry and microscopy, based on the simultaneous measurements of the intrinsic fluorescence of nicotinamide adenine dinucleotide (NADH) and nicotinamide adenine dinucleotide phosphate (NADPH), have been also demonstrated^7^ for studying cellular energy metabolism.

Among the several existing ratiometric techniques, Forster resonance energy transfer (FRET)^8^^–^^10^ and fluorescence polarization (FP)/fluorescence anisotropy (FA)^11^^,^^12^ are probably among the most widespread. Ratiometric determination of the efficiency of fluorescence (FRET) is typically used to analyze protein clustering and conformation. FA measurements instead are utilized for measuring equilibrium binding constants, molecular interactions, and enzymatic activity.

The major problem associated with ratiometric techniques resides in the fact that the fluorescence signal is always affected by signal fluctuations, which considerably worsen the ratio estimation as the light collected by the detector diminishes. This effect is particularly relevant in all those cases where the fluorescence imaging process is characterized by a low efficiency and can be only partially compensated by longer integration times. As a consequence, the ratio formed in the presence of weak signals can be highly inaccurate due to fluctuations in the ratio, which result in compounding error propagation^9^^,^^10^ and wild ratio estimates. Temporal or spatial filtering could be effective in mitigating these effects but at the expenses of the temporal or spatial resolutions,^13^ particularly when dealing with dynamic imaging. Also other sophisticated approaches based on probabilistic methods such as multivariate statistical optimization^13^ or maximum likelihood estimation could be utilized^14^ to the same end.

In this manuscript, we focus on how we can address the problem of noise mitigation in FA imaging microscopy using an image acquisition strategy that integrates high dynamic range (HDR) imaging with FA microscopy. The underlying strategy to increase the FA image information content consists in acting at the first stages of the signal acquisition chain, extending the dynamic range of the single FP components rather than to postprocess individual images with low signal-to-noise ratio (SNR) or the noisy derived ratiometric quantity.

## 2 Theory

**Fig. 1:**
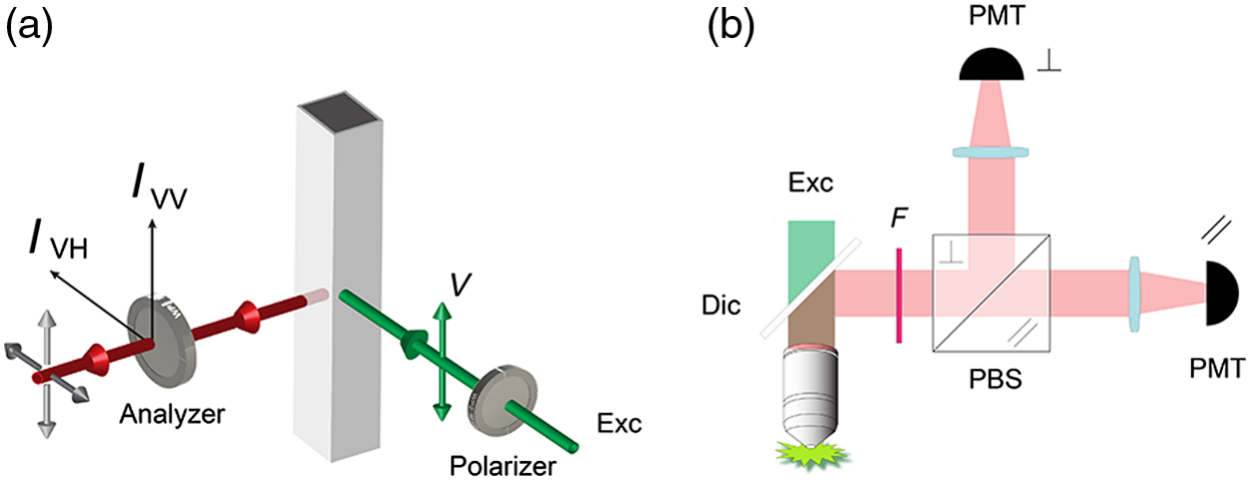
FA principle. (a) After excitation with a vertically polarized light, fluorescence emission is measured through an analyzer oriented parallel ($I_{\mathrm{VV}}$) and perpendicular ($I_{\mathrm{VH}}$) to the excitation light. (b) FA microscopy images are obtained measuring simultaneously, in a T-format scheme, the two orthogonal components of the fluorescence emission.

The underlying principle of FA^15^ resides in the fact that when a sample composed of randomly distributed molecules is illuminated with vertically polarized light, only dye molecules with transition moments oriented along this axis will be more likely excited, resulting in a nonrandom distribution of the excited molecules transition moments (photoselection process). The probability of the excitation depends on the angle θ between the light excitation vector and the dye transition moment, following a $\cos^{2}\;\vartheta$ law. The presence of the resulting uneven fluorescence intensities along the coordinate axes^16^ and the angular relation that exists between the absorption and the emission directions can be characterized with the FP parameter p defined as

$$p=\frac{I_{\mathrm{VV}}-I_{\mathrm{VH}}}{I_{\mathrm{VV}}+I_{\mathrm{VH}}},$$ (1)

where $I_{\mathrm{VV}}$ and $I_{\mathrm{VH}}$ refer to the vertical and horizontal components of the recorded fluorescence [Fig. 1(a)] with respect to a vertically polarized excitation (V).

The FP is an “intensive property”, i.e., independent on the amount of fluorophore,^16^ because both numerator and denominator are themselves proportional to the fluorophore concentration. It is also insensitive to inner-filter effects^17^ and while the terms FP and FA are interchangeably used, the concept of FA, defined as

$$r=\frac{I_{\mathrm{VV}}-I_{\mathrm{VH}}}{I_{\mathrm{VV}}+2I_{\mathrm{VH}}},$$ (2)

is more appropriate, because it more correctly describes the radiation field rather than the incoming light. Another benefit of expressing this phenomenon in terms of FA is that it is additive in its form, allowing to easily obtain an immediate resolution of freely rotated and bounded fluorophores.^16^

In the absence of depolarizing processes, the values of the fundamental FA may vary with wavelengths, fluorophores, and types of measurement (1- versus 2-photon) and are limited within specific ranges. In solutions or intracellular environments instead, the presence of depolarization effects such as the rotational diffusion can remove any preferential emission direction^15^ and the observed FA will be directly related to the apparent molecular weight, which can change upon complex formation (Perrin law^18^^,^^19^). As a result FA imaging can be successfully used to study protein–protein interactions,^20^^,^^21^ to resolve dissociation constants,^22^^,^^23^ and to enable high throughput screening of small molecule libraries for drug discoveries.^24^

Recently, we have shown how FA microscopy live-cell imaging can be successfully used to measure and map drug–target interactions in real time at subcellular resolution.^16^^,^^25^^–^^29^ In this modality, individual FP images are typically acquired in a T-format scheme [Fig. 1(b)] with two detectors registering the parallel ($I_{\mathrm{VV}}$) and perpendicular ($I_{\mathrm{VH}}$) components of the fluorescence intensity signal, and the FA images are then obtained on a pixel-by-pixel basis. Because they are derived from Eq. (2), FA images are inherently intensity ratiometric quantities as well, independent of the total intensity of the sample.^15^

Although FA images provide extremely valuable information, the ratio involved in their computation makes them very sensitive to the noise. As a result, they are prone to exhibit low contrast and in turn be poorly informative from an imaging point of view.

**Fig. 2:**
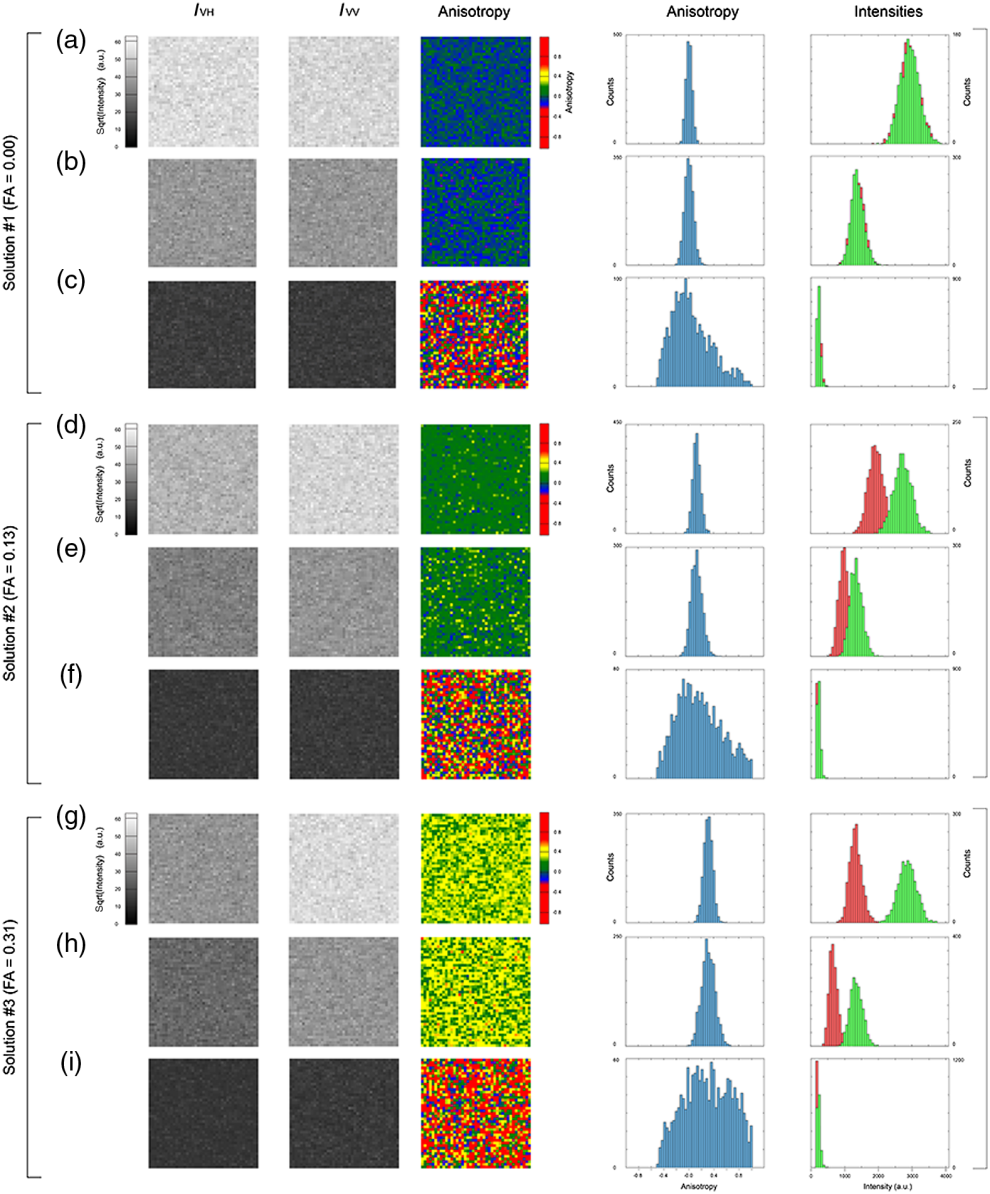
Images of (a)–(i) three dye solutions (FITC with varying concentration of glycerol) presenting different values of anisotropy (0.00, 0.13, and 0.31). The first and second columns correspond to the two linearly polarized orthogonal states of polarization detection channels. The third column corresponds to the calculated anisotropy, as obtained from the fluorescence intensity measurements after background subtraction. Here the background value is computed as the average across a dark image collected on each individual FP channel. The fourth column corresponds to the FA histogram. The fifth column indicates the signal distribution of the two orthogonal channels (red, perpendicular; green, parallel). The intensity is displayed using a nonlinear scale to visually emphasize the noise contributions.

We illustrate this concept in Fig. 2. Here, FA imaging was performed on three homogeneous solutions [Figs. 2(a)–2(c), 2(d)–2(f), and 2(g)–2(i)] presenting different anisotropy values ranging from low to high (0.00, 0.13, and 0.31, respectively).

For each solution, three different images at different values of excitation intensity (high, medium, and low) were taken. The goal was to mimic different possible imaging conditions as normally found in biological samples, both in terms of FA and fluorescence intensity signal, and to analyze their signal distributions under different conditions.

The first two columns, which correspond to the images of the fluorescence intensity with polarization components perpendicular ($I_{\mathrm{VH}}$) and parallel ($I_{\mathrm{VV}}$) to the excitation one [Fig. 1(b)], show that an increase in the FA values results in lower signal detected on the first channel (orthogonal component). Also the lower the fluorescence signal, the lower the SNR of both intensity, and anisotropy images will be, with a spreading of the FA histogram (Fig. 2, column 4) inversely related to the fluorescence intensity (Fig. 2, column 5). It is, therefore, evident how increasing the number of collected photons not only allows for better fluorescence images, but it is also beneficial at reducing the deviation of the actual measurement of the FA from the expected value.

Another consideration to make is that a broadening in the FA distribution, as a function of noise level, leads to a loss of image contrast. This is crucial because the performance of algorithmic-based image analyses, which exploit for instance segmentation and/or feature tracking, will be dramatically influenced by the loss of image quality, therefore preventing accurate image feature analysis (e.g., differentiating FA values between different compartments of a cell over time).

**Fig. 3:**
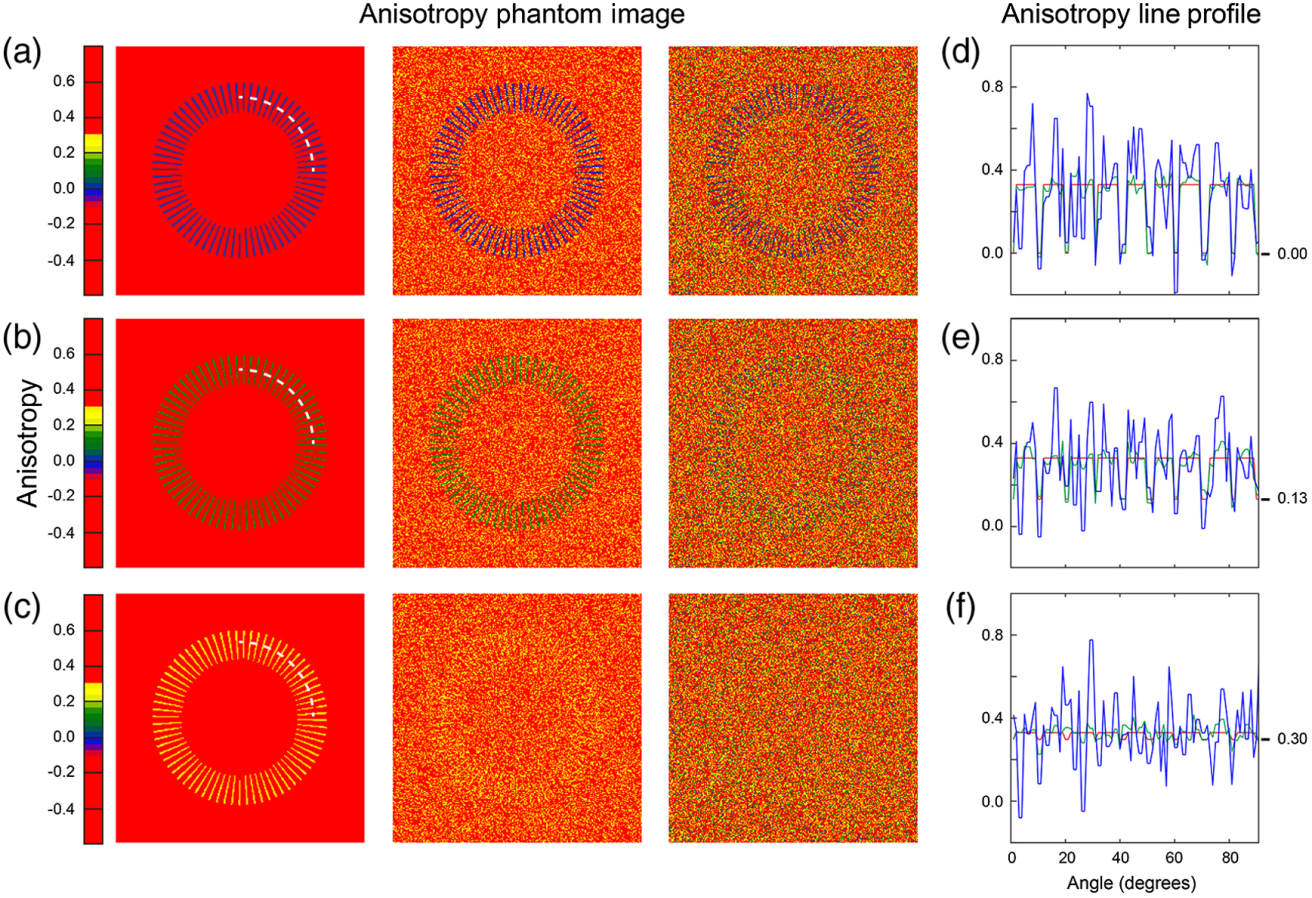
Anisotropy images of a synthetic phantom generated with signal and noise parameters (Poisson noise and dark noise) as present under typical acquisition imaging sessions. The average background is kept constant at an FA value of 0.33. Inner ring radial features are progressively changing their FA value from (a) 0 to (b) 0.13 and (c) 0.30. First column, phantom in the absence of noise. Second and third columns, phantoms presenting decreasing values of total intensity. (d)–(f) FA line profiles taken along the white dashed lines in (a)–(c): noise-free (red), high SNR (green), and low SNR (blue).

To better illustrate this concept, we consider here a synthetic phantom (Fig. 3) comprised of a ring-shaped element embedded within a uniform distributed background kept at FA value of 0.33. The first column (top to bottom) shows a noise-free phantom with inner ring radial structures presenting a value of anisotropy progressively changing from 0.00 [Fig. 3(a)], to 0.13 [Fig. 3(b)], and 0.30 [Fig. 3(c)]. This first column is used as a reference (i.e., it is the image presenting the maximum possible contrast). The other two columns are simulated images using counts as typically obtained during regular imaging sessions with pixel intensity levels ranging in the order of the thousands (column 2) to the hundreds (column 3) counts, together with their associated Poisson noise. In both simulations, we used the dark noise levels associated to the detectors at the typical imaging voltage settings.

From the data, we clearly see that high SNR images yield to better contrast as evidenced also in their associated line profiles [Figs. 3(d)–3(f)]. An extension of the dynamic range of the single polarization components of the fluorescence intensity can, therefore, lead to a reduced effect of noise and a better contrast on anisotropy imaging.

## 3 Methodology

Among the several sources of noise that affect the quality of the acquired fluorescence intensity components and consequently the derived FA, the main one is the photon noise related to the quantal nature of light.^30^ To increase the quality of the individual polarization measurements, we can make use of *ad hoc* signal acquisition schemes, which rely on the modeling of the light signal as a Poisson process. Interestingly, for a Poisson random distribution, the expected value is equal to its variance.^16^ By defining the SNR for a Poisson variable as its first moment divided by the square root of its second moment, it is possible to derive that an increase in signal intensity helps originate better quality images. Therefore, according to this depiction, the light noise reduces its contribution proportionally to the expected value, as the number of photons collected by the detector increases, and FA imaging will be severely impacted at low count rates.

Two straightforward ways we have at our disposal and that allow for an increase in the total number of photons consist in either increasing the excitation laser power or alternatively augmenting the detection integration time. Although these two strategies are regularly used in microscopy, they may unfortunately lead to the saturation of the detectors. This is particularly troublesome because for biological imaging the typical intrascene dynamic range (IDR), which is determined by the distribution and/or concentration of protein expression or fluorophores, can be much larger than the detector’s dynamic range. If this is the case, the acquired images will then present different regions with fluorescence intensities saturated or below the background. The result is that we obtain a loss of the image informative content effectively reducing the detectors’ bit depths,^31^ with the impossibility to differentiate among different structures and giving rise to severely impaired image quality.

The impact is even more dramatic when calculating a ratiometric quantity such as the FA, where two separate measurements of the individual FP components are acquired. Another consideration to make is that signal distribution in FA images varies not only within single images but also among the two separate orthogonal channels, with its impact more severe when higher values of anisotropy are present (i.e., the orthogonal components tend to get smaller and therefore noisier than the parallel one).

So far, different software or hardware-based approaches have been developed to extend the dynamic range of optical imaging detectors, offering both the resolution and the sensitivity necessary for performing imaging at the cellular and subcellular level.^32^ Recently, we have introduced multiexposure HDR imaging for confocal and two-photon laser scanning microscopy,^32^ extending this strategy in the biomedical imaging field. The technique permits to acquire both dark and bright image areas within a field of view with a proper SNR and, at the same time, to avoid image saturation, originating composite 32-bits HDR image reconstructions.

By integrating HDR imaging with FA and parallelizing the HDR acquisition scheme for each individual polarization channel, we demonstrate here that HDR-based FA images with highly improved SNR can be obtained. To this aim, dual-channel FP images [low dynamic range (LDR)] with different degrees of signal levels were taken and fused together throughout an algorithm^32^ that gives rise to single unsaturated images characterized by a dynamic range exceeding the one present in normal acquisitions.

**Fig. 4:**
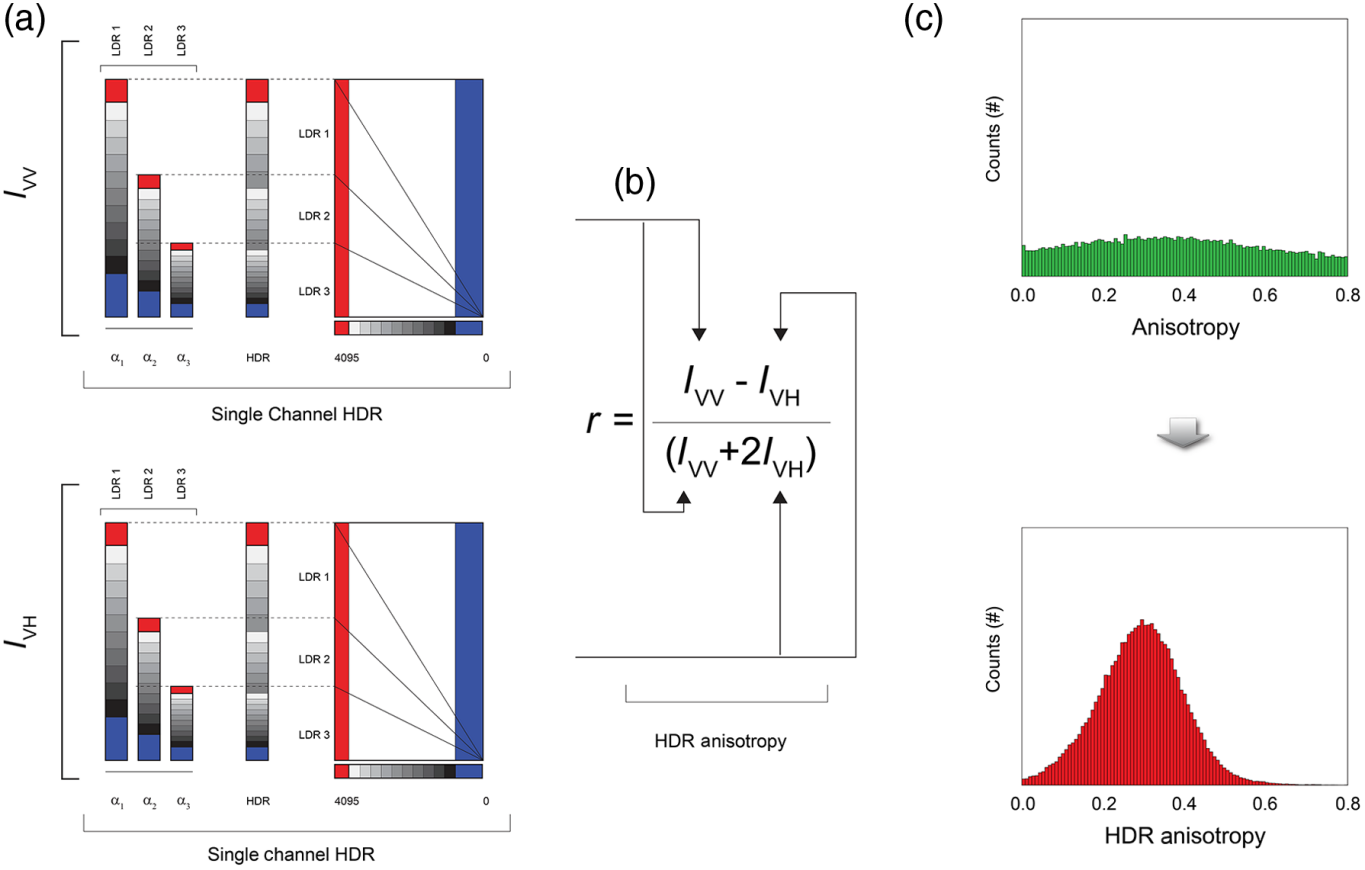
Principle of HDR-based FA imaging. (a) LDR images (LDR1, LDR2, and LDR3) for both polarization components of the fluorescence intensity ($I_{\mathrm{VV}}$ and $I_{\mathrm{VH}}$), and progressively saturated in intensity, are acquired to produce two final images with extended dynamic ranges. (b) An HDR-based FA image is then calculated, with a final increased SNR. (c) Pixel-by-pixel FA histogram before (green) and after (red) range extension.

Specifically, each LDR image was taken by varying a parameter α that regulates the amount of light to which the detector was exposed [Fig. 4(a)].^32^ Commonly, two or three images (image series) are enough to achieve satisfactory results.

Because the algorithm that allows an image series to be fused into an HDR image is identical over both orthogonal channels, we here limit its explanation to one channel only without any lack of generality. Also because the actual image fusion algorithm is iterated over all those pixels that belong to the same position within the image series, it is possible to fully parallelize the pixel blending procedure.

The HDR algorithm first applies a background subtraction to each pixel $P_{ij}$ leading to a new value $P_{ij}^{\prime }$, where i is the index of the pixel position within the j’th image of the series (consisting of N images). In general, $P_{ij}^{\prime }$ is then transformed through a function that combines both the detector response and the modulator factor used to achieve different levels of intensities for the same pixel location. Therefore, each image pixel $P_{ij}^{\prime }$ is mapped into a new pixel $Q_{ij}$, according to $Q_{ij}=f(r,P_{ij}^{\prime },\alpha_{j})$, where f is, in general, a nonlinear function whose parameters are the detector response r, the pixel value $P_{ij}^{\prime }$ and the modulator factor $\alpha_{j}$, which proportionally correlates with the amount of signal at the detector. Because the fluorescence signal is heterogeneous over a large sample field of view, it is recommended to vary the excitation power depending on the overall field of view maximum signal, by way of a circular neutral density filter wheel (alternatively by using a set of optical density filters arranged on an automatic filter wheel or manually adjusting the power) and empirically estimate the f function by solving a linear least square problem.^33^ Alternative ways could be also used, for example, by changing the pixel integration time, the PMTs voltages, or by adding additional recording channels in order to perform real-time HDR recording of each single orthogonal FP components in a similar fashion as discussed in details in Ref. 32. Each method is valid and it comes with its pros and cons (see Table 1). If the overall system response can be considered approximately linear, the formula simplifies to $Q_{ij}=P_{ij}^{\prime }/k_{j}$, where k is the amplification of the signal due to the increase of the modulator factor. Once all $Q_{ij}$ are computed, each HDR image pixel ($I_{\mathrm{HDR},i}$) is obtained through a weighted average^32^ as follows:

$$I_{\mathrm{HDR},i}=\frac{\sum_{j=1}^{N}Q_{ij}w(P_{ij}^{\prime })}{\sum_{j=1}^{N}w(P_{ij}^{\prime })},$$ (3)

where w is a triangular-window weighting function with a peak in the middle of the dynamic range used to acquire the LDR images.^33^

**Table 1:** The pros and cons of different ways in which the HDR parameter α can be modified in order to acquire an image series consisting of images with increasing signal intensities.

Parameter α	+	−
Integration time	Easy implementation	Sequential acquisition, longer acquisition time, and possible artifacts
PMT voltages	Easy implementation	Sequential acquisition, longer acquisition time, possible artifacts, noise variations, and different PMT calibration curves
Excitation power	Easy implementation and possible control with insertion of neutral density filters or controlling directly the laser power	Sequential acquisition, longer acquisition time, and possible artifacts
Real-time HDR using a beam splitter^32^	Fastest acquisition rates, simple calibration, fixed laser power settings, and no artifacts	Increased complexity of the setup and fix splitting ratio

Once the single orthogonal FP components images were fused together, the HDR-based FA image was directly obtained by way of Eq. (2) on a pixel-by-pixel basis using the correspondent pixel values from the two previously calculated HDR FP images as given in Eq. (3).

Because HDR images are characterized by a higher SNR, pixel-by-pixel histograms of the final FA images will tend to present a substantial reduction in their width as compared to the original ones [Fig. 4(c)].

## 4 Experiments and Results

To demonstrate the feasibility of our method on a biological sample, we chose a specimen presenting an extended range of both fluorescence intensity and FA on separate cellular compartments. Specifically, we used a prepared microscope slide (FluoCells, Invitrogen) containing fixed bovine pulmonary artery endothelial (BPAE) cells. MitoTracker Red CMXRos was used to stain the mitochondria, whereas F-actin was stained with Alexa Fluor 488 phalloidin, and the nuclei were counterstained with DAPI.

HDR-based FA imaging was implemented in two-photon using a commercially available imaging system (FV1000, Olympus, USA) with a tunable MaiTai DeepSee Ti:sapphire pulsed laser (Spectra Physics). A Glan–Thompson polarizer combined with a half-wave plate was used to polarize the excitation light, while the emitted light was collected in a nondescanned mode. Fluorescence images were acquired in a T-format scheme [Fig. 1(b)]. The fluorescence signal was separated into two orthogonal linearly polarized states and simultaneously detected by two separate photomultiplier tubes. The orthogonal HDR FP images and the HDR-based FA images were then obtained on a pixel-by-pixel basis as described in Sec. 3. The parameter α for the HDR fusion algorithm was modulated by controlling the laser power on the sample. The acquisition parameters are given in Table 2.

**Table 2:** The acquisition parameters for the orthogonal FP components and the GT images.

Channels	Integration time (μs)	Averaging	PMT voltage (V)	Estimated κ’s see Eq. (3)
$I_{\mathrm{VH}}$	20	None	581	1-3.16-15.18
$I_{\mathrm{VV}}$	20	None	598	1-3.16-15.18
GT $I_{\mathrm{VH}}$	20	40×	581	None
GT $I_{\mathrm{VV}}$	20	40×	598	None

**Fig. 5:**
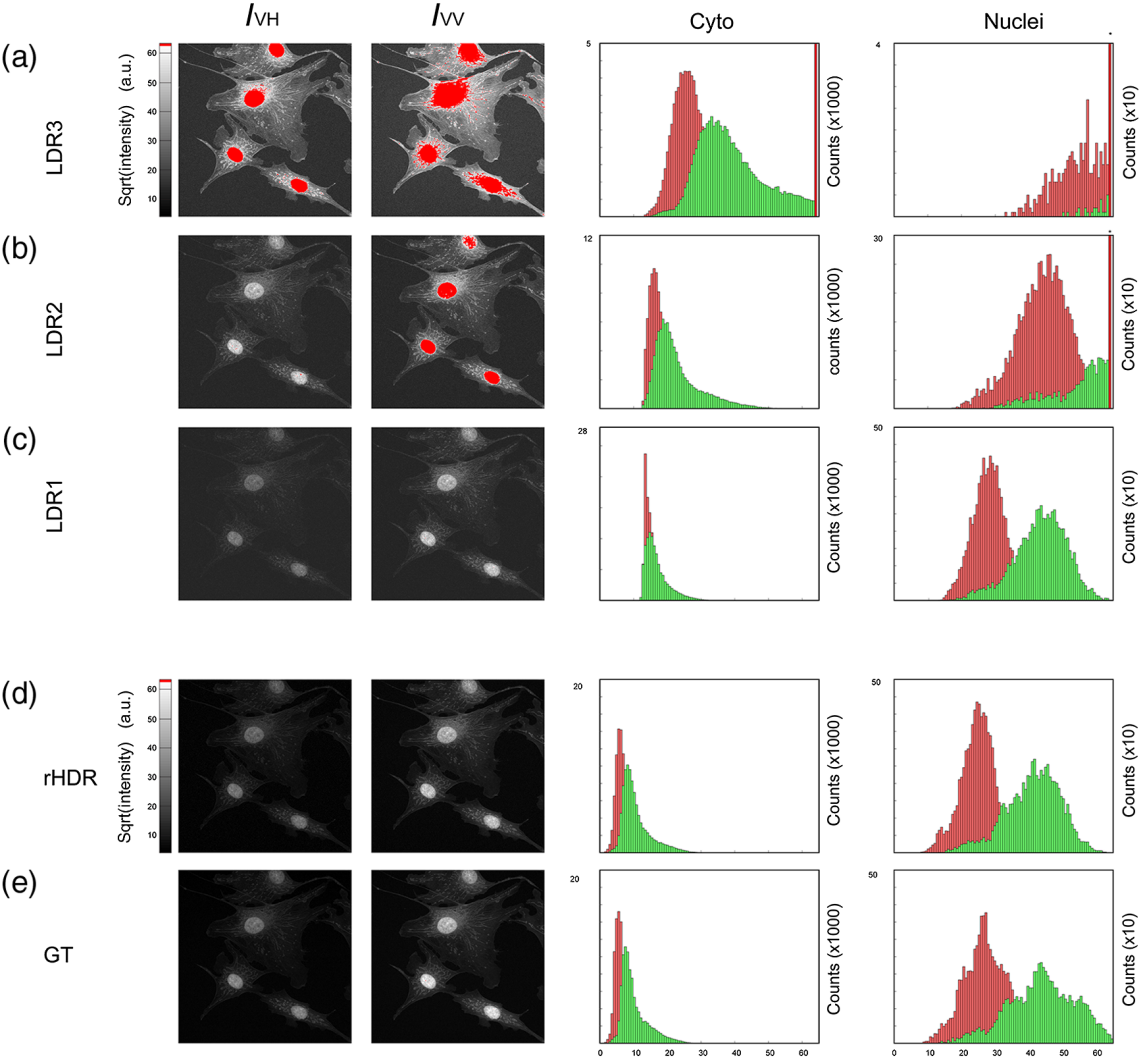
(a)–(c) LDR, (d) HDR, and (e) GT images of the orthogonal FP components, along with their associated fluorescence intensity histograms. The first and the second columns correspond from top to bottom to LDR3, LDR2, LDR1, HDR, and GT images of the orthogonal and parallel components of the fluorescence intensity ($I_{\mathrm{VH}}$ and $I_{\mathrm{VV}}$). The red colored pixels, indicating signal saturation areas, were discarded from the HDR fusion algorithm. The third and fourth columns show the fluorescence intensity histograms of the cytoplasmic and nuclear regions, respectively ($I_{\mathrm{VH}}$ channel, red; $I_{\mathrm{VV}}$ channel, green). Histogram bars with an overlaid asterisk indicate range out of scale. HDR and GT images are remapped, with background subtraction.

The FP components images of the BPAE cells were recorded on the two separate PMTs, progressively increasing the excitation laser intensity (LDR1, LDR2, and LDR3) such that all areas within the sample could be recorded with satisfactory signal levels [Figs. 5(a)–5(c)].

**Fig. 6:**
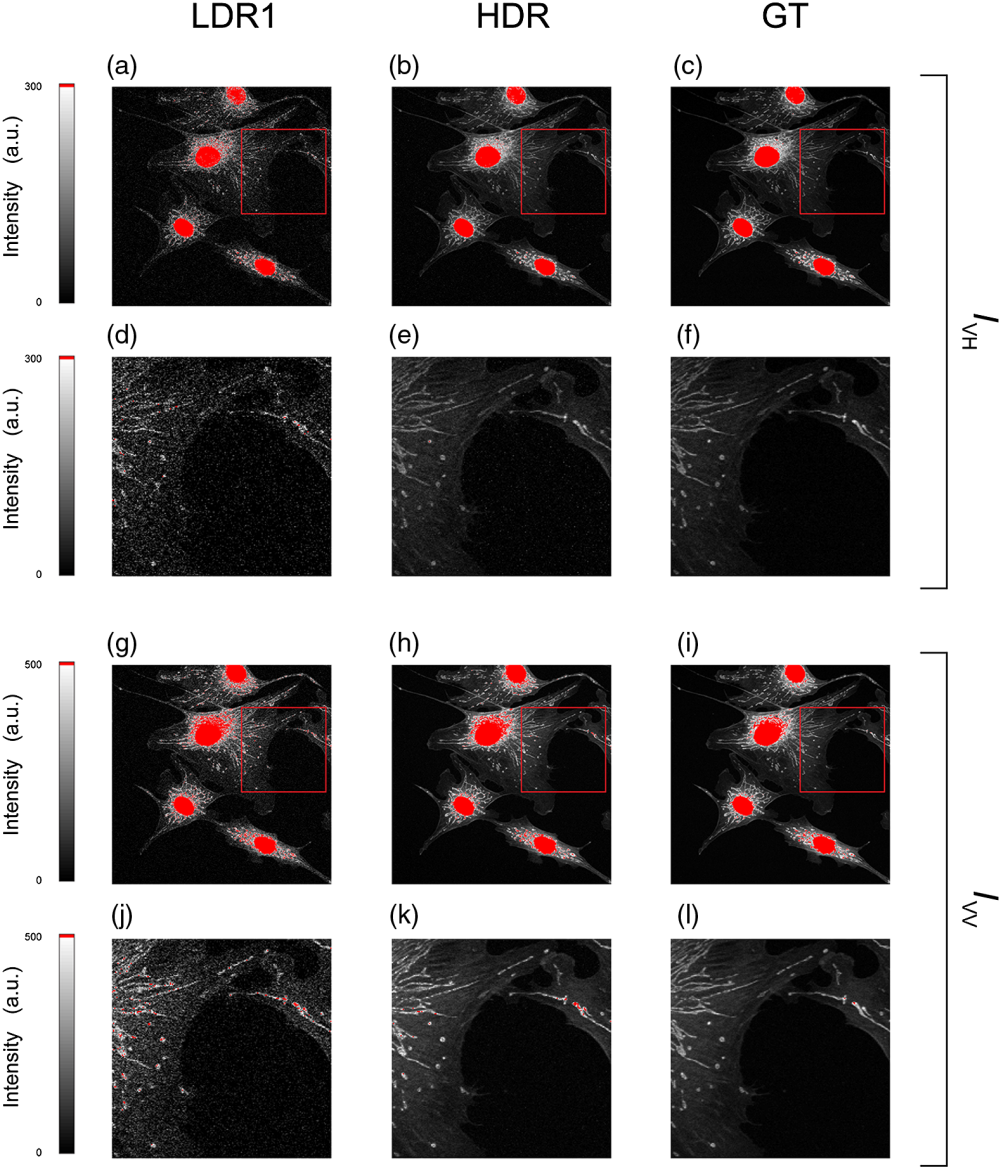
Comparison of LDR, HDR, and GT images of the orthogonal FP components shown in Fig. 5 and visualized in a linear scale at low counts. (a)–(f) $I_{\mathrm{VH}}$ channel and (g)–(l) $I_{\mathrm{VV}}$ channel. The images in (d)–(f) and (j)–(l) refer to the magnified area in the red box in (a)–(c) and (g)–(i).

In the first two columns of Figs. 5(a)–5(c), images of the fluorescence intensity with the polarization components perpendicular ($I_{\mathrm{VH}}$) and parallel ($I_{\mathrm{VV}}$) to the excitation polarization are shown. Different structures in the field of view are present within the entire dynamic range of the image, and their associated intrinsic noise makes it difficult to clearly resolve them all in a single LDR image (red colored pixels indicate signal saturation areas). Using the individual LDR images obtained by progressively increasing the excitation intensity (α parameter), single HDR FP images are obtained through the HDR fusion algorithm as described in Sec. 3. Because the individual HDR FP images exhibit a wide range of values that cannot be represented on normal monitors, remapped HDR unsaturated images are shown [Fig. 5(d)]. The images are clearly characterized by a dynamic range exceeding the one present in the LDR FP images. Ground truth (GT) images of both FP components are obtained by averaging LDR images over 40 acquisitions and given as a reference [Fig. 5(e)]. A comparison between the individual FP images in Fig. 5(d) with the ones in Fig. 5(e) clearly indicates how HDR-based FP microscopy imaging allows us to achieve high-quality fluorescence images that are comparable with our GT. The improvement in the HDR-based FP microscopy images versus the LDR ones is also evident when comparing them in a linear scale at low counts, where the noise is predominant in the LDR ones (Fig. 6).

This emerges also when analyzing the HDR FP intensity histograms, where signal distribution profiles more accurately approximate the GT reference ones.

**Fig. 7:**
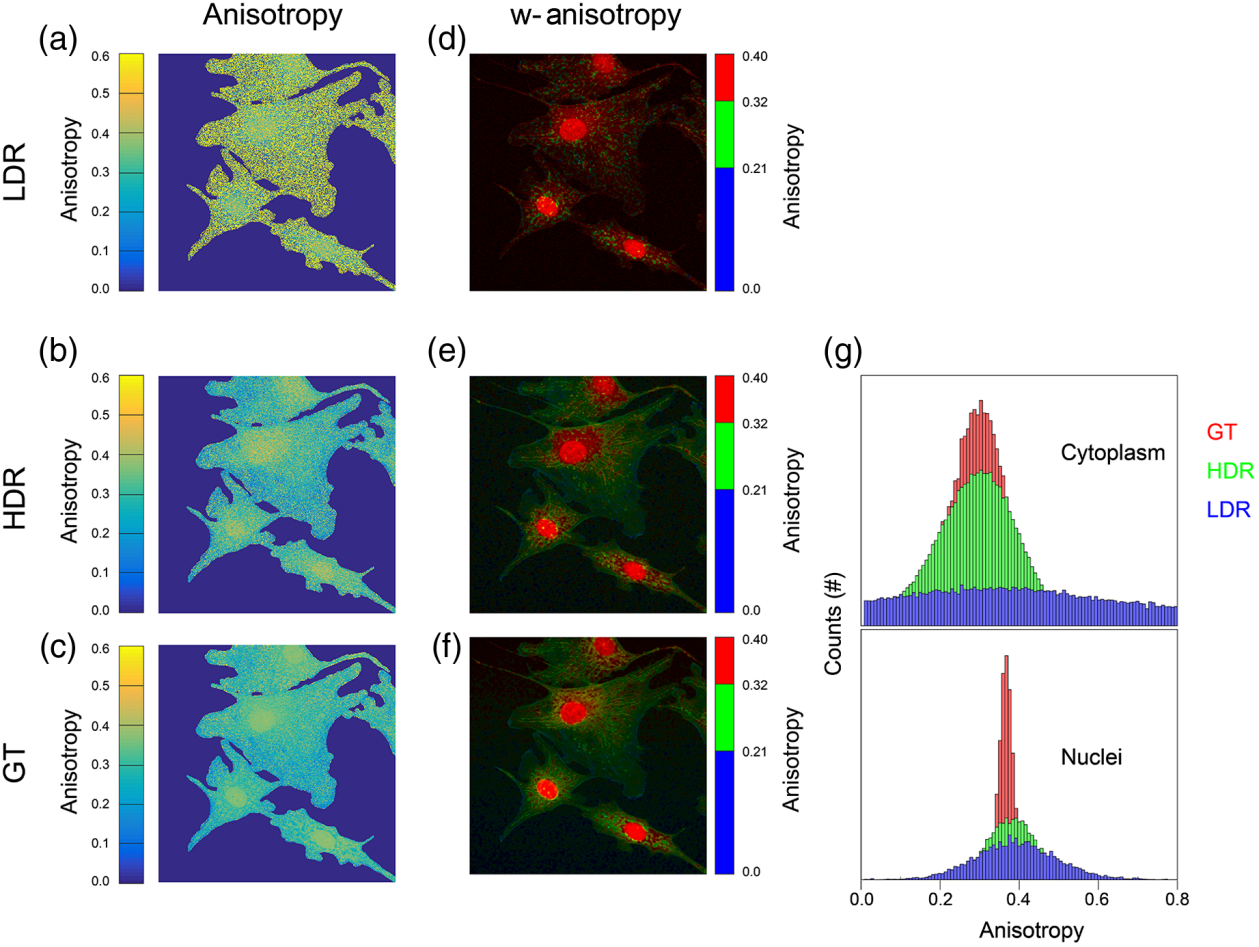
HDR-based FA imaging: (a) LDR FA, (b) extended dynamic range (HDR)-based FA, (c) GT FA, and (d)–(f) weighted FA images. (g) Comparison between the pixel-by-pixel histogram distributions of the FA values within the cytoplasmic (top) and nuclear (bottom) compartments for the LDR, HDR-based, and GT cases.

HDR-based FA images [Figs. 7(a)–7(c)] were then calculated making use of the calculated individual HDR FP images [Figs. 4(a) and 4(b)].

Due to the limited acquisition range, the FA images [Figs. 7(a) and 7(d)] calculated on the unsaturated LDR1 images [Fig. 5(c)] result to be very noisy. This makes it extremely difficult to resolve the nuclear area from the cytoplasmatic ones as well as to analyze the distribution of the FA signal within the cytoplasm itself.

A direct visual comparison with the HDR-based FA image [Fig. 7(b)] shows that the extended dynamic range present in the individual HDR FP components increases the accuracy of the calculated HDR-based FA image.

The resulting image is similar in quality and signal content with the GT FA image [Fig. 7(c)] obtained by extensive time averaging during the individual FA components acquisition [Fig. 5(e)].

This also emerges clearly when considering the weighted FA images [Figs. 7(d)–7(f)]. In these, the FA information is color encoded and statistically weighted by the total intensity of the fluorescence signal. This display modality is helpful for combining both functional (FA) and morphological (fluorescence) information into a single image, allowing fine structures, like mitochondria, to be adequately highlighted. As clearly evident by a direct comparison with the GT equivalent [Fig. 7(f)], HDR-based FA images [Fig. 7(e)] yield to high-quality images, whereas standard FA imaging [Fig. 7(d)] tends to assign wrong anisotropy values at all those structures within the field of view that present low fluorescence intensity (mitochondria).

It is also interesting to analyze the pixel-by-pixel histograms of the derived FA, as calculated within the cytoplasm and the nuclei regions for both LDR, HDR-based and GT images [Fig. 7(g)]. From a comparison between the distributions, it is evident how for both compartments, the HDR-based FA histograms (green bars) resemble more the GT (red bars) ones, while the FA signal distributions in the LDR (blue bars) tend to distributes over a substantially higher range of values, giving rise to incorrect assigned values.

## 5 Conclusions

In conclusion, we have presented an HDR-based FA imaging modality, as a method for improving image quality in FA microscopy.

Because the noise is intrinsically entangled to the light signal and because it is not uncommon for fluorescence images to suffer from low SNR, particularly in *in vivo* imaging settings, FA image degradation can be quite severe and strategies to overcome, or at least, ameliorate image degradation are in need. By integrating HDR imaging with FA microscopy, we have demonstrated that FA images obtained by exploiting *ad hoc* acquisition schemes that extend the dynamic range of the individual polarization channels outperform standard FA imaging obtained under standard dynamic range imaging conditions. In fact, the mitigating effect on the photon noise introduced by the proposed technique directly impacts the image quality of the FP components, which in turn positively reflects on the ratiometric measurements of which FA is an instance.

Because our imaging modality can be easily integrated and performed on any commercially available microscopes, it could help to facilitate FA imaging particularly of biological structures where typical IDR are larger than the detectors dynamic range.

Our imaging modality could also be extended to other ratiometric quantities as well, allowing to easily assess other functional information.
